# Construction and validation of a clinical predictive nomogram for intraductal carcinoma of the prostate based on Chinese multicenter clinical data

**DOI:** 10.3389/fonc.2022.1074478

**Published:** 2022-12-15

**Authors:** YunKai Yang, Wei Zhang, LiJun Wan, ZhiLing Tang, Qi Zhang, YuChen Bai, DaHong Zhang

**Affiliations:** ^1^ Urology & Nephrology Center, Department of Urology, Zhejiang Provincial People’s Hospital, Hangzhou, Zhejiang, China; ^2^ The 2nd Clinical Medical College, Zhejiang Chinese Medical University, Hangzhou, Zhejiang, China; ^3^ Zhejiang Provincial People’s Hospital, Qingdao University, Shandong, Qingdao, China; ^4^ Department of Urology, Quzhou People’s Hospital, Quzhou, Zhejiang, China; ^5^ Department of Urology, Jiaxing Second People’s Hospital, Jiaxing, Zhejiang, China

**Keywords:** PCa, IDC-P, predictive nomogram, biochemical indicators, obesity

## Abstract

**Introduction:**

Intraductal carcinoma of the prostate (IDC-P) is a special pathological type of prostate cancer that is highly aggressive with poor prognostic outcomes.

**Objective:**

To establish an effective predictive model for predicting IDC-P.

**Methods:**

Data for 3185 patients diagnosed with prostate cancer at three medical centers in China from October 2012 to April 2022 were retrospectively analyzed. One cohort (G cohort) consisting of 2384 patients from Zhejiang Provincial People’s Hospital was selected for construction (Ga cohort) and internal validate (Gb cohort)of the model. Another cohort (I cohort) with 344 patients from Quzhou People’s Hospital and 430 patients from Jiaxing Second People’s Hospital was used for external validation. Univariate and multivariate binary logistic regression analyses were performed to identify the independent predictors. Then, the selected predictors were then used to establish the predictive nomogram. The apparent performance of the model was evaluated *via* externally validated. Decision curve analysis was also performed to assess the clinical utility of the developed model.

**Results:**

Univariate and multivariate logistic regression analyses showed that alkaline phosphatase (ALP), total cholesterol (TC), triglycerides (TG), high-density lipoprotein (HDL), prostate specific antigen (PSA) and lactate dehydrogenase were independent predictors of IDC-P. Therefore, a predictive nomogram of IDC-P was constructed. The nomogram had a good discriminatory power (AUC = 0.794). Internal validation (AUC = 0.819)and external validation (AUC = 0.903) also revealed a good predictive ability. Calibration curves showed good agreement between the predicted and observed incidences of IDC-P.

**Conclusion:**

We developed a clinical predictive model composed of alkaline phosphatase (ALP), total cholesterol (TC), triglycerides (TG), high-density lipoprotein (HDL), prostate specific antigen (PSA) and lactate dehydrogenase (LDH) with a high precision and universality. This model provides a novel calculator for predicting the diagnosis of IDC-P and different treatment options for patients at an early stage.

## Introduction

Globally, clinical incidences for prostate cancer (PCa) are increasing at a rate of 4% to 6% per year, and it accounts for 7.3% of all cancer cases. In over one-half (112 of 185) of the countries of the world, it is the most frequently diagnosed cancer among men (1). Intraductal carcinoma of the prostate (IDC-P) is a biologically aggressive form of PCa that is characterized by the proliferation of malignant cells within prostatic ducts and acini (2, 3). Clinically, IDC-P has poor prognostic outcomes, including higher pathological stage, high Gleason score, larger tumor size, and a high risk of lymphatic metastasis. Moreover, compared to the conventional prostatic acinar adenocarcinoma (PAA), IDC-P has a high risk of clinical biochemical recurrence, metastasis, as well as a poorer progression-free survival and overall survival outcomes (4, 5). The current therapeutic options for IDC-P include neoadjuvant endocrine therapy, postoperative chemotherapy consolidation, and radiotherapy among others (4–6).

Pathologically, IDC-P is often accompanied by other types of prostate cancer (7–10), including prostate adenocarcinoma and squamous cell carcinoma of the prostate. In addition, it has highly comparable histological characteristics with high Gleason-classified prostate malignant tumors. Since it is difficult to directly diagnose IDP-C using a prostatic biopsy, it can easily be misdiagnosed clinically. Patients who are diagnosed with distant metastases or with serious underlying diseases often do not have the opportunity for surgeries (11, 12). As a result, determination of the pathology of many patients by paraffin sections is challenging. Therefore, an early and accurate diagnosis of IDC-P is crucial.

Hematological parameters include clinical indicators that are easily accessible. They can be used to detect the contents of various ions, sugars, lipids, proteins, various metabolites of enzymes, and hormones in the blood. These indices reflect the condition of the internal environment of the body. The relationship between blood biochemical parameters and IDC-P is yet to be elucidated (13–17). We constructed a predictive model that is based on biochemical indicators to assist in the diagnosis of IDC-P diagnosis.

## Materials and methods

### Study design and participants

This was a multi-center retrospective study involving 3185 PCa patients from three independent regional medical centers in China was conducted. Data for 2384 patients had been admitted to Zhejiang Provincial People’s Hospital from October 2012 to April 2022 were collected, labeled as the G cohort, and divided into two groups for construction of the nomogram (Ga cohort; n=1556) and for internal validation (Gb cohort; n=828).

In addition, data for 344 patients who had been admitted to Quzhou People’s Hospital from April 2014 to April 2022 and 430 patients who had been admitted to Jiaxing Second Hospital from June 2013 to April 2022 were collected and labeled as the I cohort, which was used for external validation. This study was approved by the institutional ethics committee(QT2022375; Ethics Approval of Quzhou People’s Hospital No. 77, 2022; JXEY-2022HXHZ040), Each participant was required to sign an informed consent before their involvement.

### Baseline data collection and processing

**Figure 1:**
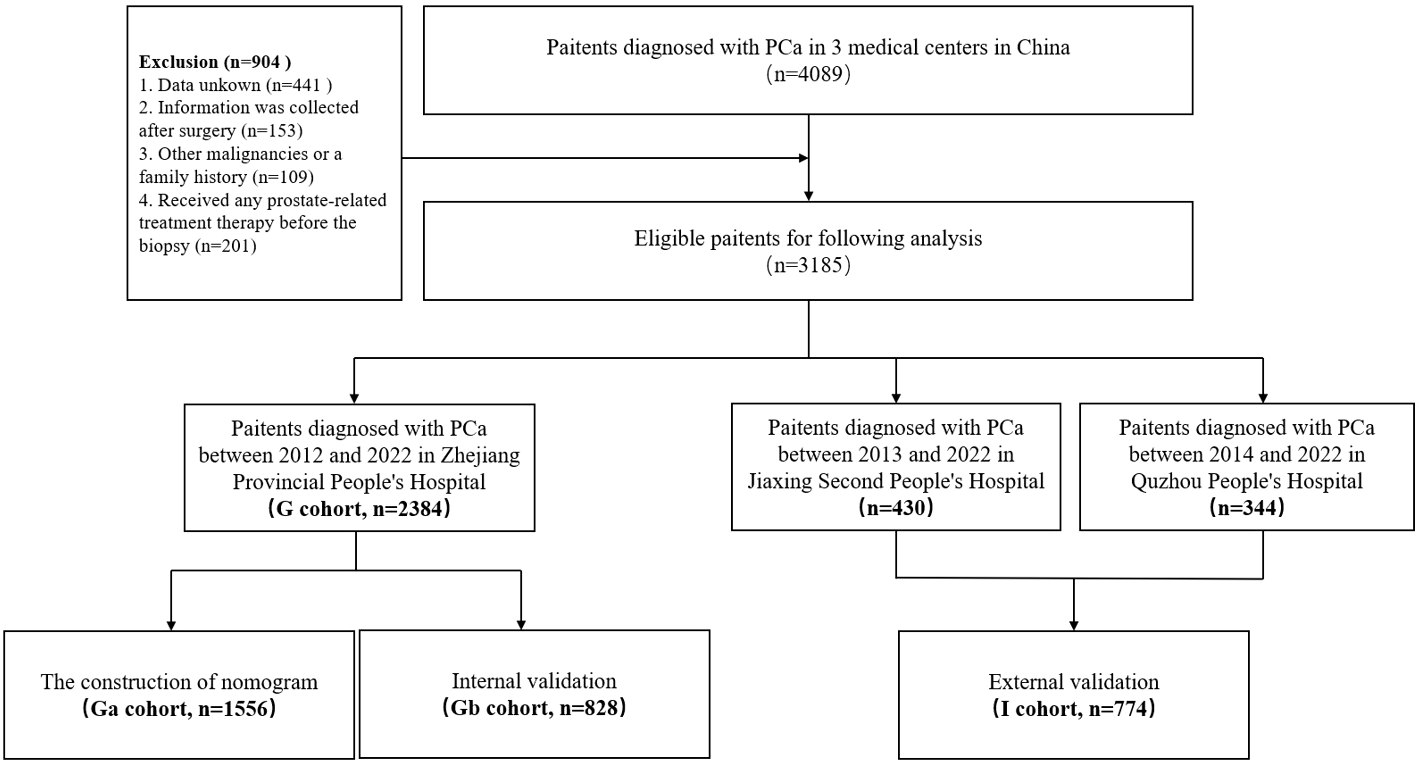
Enrollment process.

The baseline clinicopathologic information, including age, body mass index (BMI, kg/m^2^), personal history (smoking, drinking, heart disease, hypertension, and diabetes), biochemical indicators (albumin, globulin, glutamic pyruvic transaminase, glutamic oxaloacetic transaminase, gamma-glutamyltransferase, alkaline phosphatase, total bilirubin, bile acids, glucse, urine creatinine, uric acid, total cholesterol, triglyceride, high-density lipoprotein, low-density lipoprotein, lactate dehydrogenase), and prostate specific antigen (PSA) were collected. All patient information (including biochemical information) was sent to the Zhejiang Provincial People’s Hospital for reviewing and grouping (**S-Table III**). A set of inclusion and exclusion criteria was formulated to screen eligible patients in the three medical centers. (1) Only patients with PCa (confirmed by biopsy pathology and postoperative paraffin biopsy results). (2) Enrolled cases with complete baseline clinicopathologic information; patients with any missing values were excluded. (3) Patient information, apart from pathological information, were collected before surgery. (4) Patients with a history of other malignancies or a family history of PCa were excluded. (5) Patients who had not received any prostate-related treatment therapy before the biopsy (**Figure 1**).

### Prostate biopsy and pathology

The final results were based on fndings from paraffin pathology. All disputed pathological results were sent to the Zhejiang Provincial People’s Hospital Pathological center for secondary pathological results review and immunohistochemical confirmation. Finally, patients whose pathological findings were not confirmatory were excluded from the study.

A positive result was defined as IDC-P or IDC-P accompanied by any other pathological type of PCa. Other pathological types of PCa, such as prostate adenocarcinoma and squamous cell carcinoma of the prostate, were considered negative outcomes.

### Model construction, validation, and statistical analysis

The G cohort was divided into two cohorts: Ga cohort was used to construct the model, while the Gb was used for internal validation. The I cohort of patients from the two other centers was used for external validation.

Baseline characteristics of patients are presented as means ± standard deviation (SD), interquartile range (IQR), range, number, and proportions. Univariate binary logistic regression analysis method was performed to compare the different variables. Variables with p ≤ 0.05 were entered into the stepwise (forward: conditional) multivariate logistic regression analysis model. Then, the odds ratios (OR) and 95% confidence interval (95% CI) were calculated. Variables with p ≤ 0.05 in the multivariate analysis were used to establish the nomogram. The performance of the nomogram was validated using the Gb and I cohort *via* the bootstrap method. Discrimination and calibration were assessed for model validation, respectively (18). Discrimination was measured using C-statistics, which is equal to the area under the curve (AUC) calculated by plotting the receiver operating characteristic (ROC) curve (19). Calibration was measured by drawing calibration curves. Statistical analysis was performed using SPSS version 26.0 and R version 4.1.1. P ≤ 0.05 was the threshold for statistically significance.

### Decision curve analysis (DCA)

In addition to providing urologists with a quantitative nomogram for diagnosis of IDC-P, a DCA curve was constructed to predict the probability of IDC-P. The clinical net benefits of our model at different threshold probabilities were quantified using the R software.

## Results

### Patient characteristics

The clinical characteristics for all study participants are shown in **Table 1**. A patient selection must strictly adhere to the above-mentioned inclusion and exclusion criteria. A total of 1556 and 828 patients were respectively included in the Ga and Gb cohorts while 774 patients were included in the I cohort respectively. The IDP-C positive rates for the Ga, Gb and I cohorts was 8.9%, 8.9% and 8.1%, respectively (**Table 1**). Standard values for all variables were defined by Zhejiang Provincial People’s Hospital, the main unit of all research centers, and are shown in the **S-Table III**. Based on the standard value, each variable is divided into high-group, standard group and low-group. In univariate analysis, the standard group was the control group while analyzing OR values (**S-Table 3**).

**Table 1:** Demographic characteristics of the patients in each cohort.

Clinical parameters	G (n=2384)		I (n=774)
	Ga (n=1556)	Gb (n=828)	
Median age, y (IQR)	70.9 (65.0-77.0)	70.6 (65.0-76.0)	70.6 (65.0-76.0)
Median BMI, kg/m2 (IQR)	23.3 (21.1-25.3)	23.3 (21.3-25.4)	23.5 (21.3-25.6)
Diabetes	199 (12.8)	78 (9.4)	89 (11.5)
Hypertension	631 (40.6)	310 (37.4)	292 (39.2)
Cardiac disease	103 (6.6)	63 (7.6)	41 (5.3)
Smoker	576 (37.0)	321 (38.8)	311 (40.2)
Drinker	496 (31.9)	283 (34.2)	250 (32.3)
IDC-P	138 (8.9)	74 (8.9)	63 (8.1)

IQR, interquartile range; BMI, body mass index; IDC-P, Intraductal carcinoma of the prostate.

A comparison of between-group differences between the internal and external validation cohorts did not reveal significant differences (**S-Table I**).

### Establishment of the nomogram

**Figure 2:**
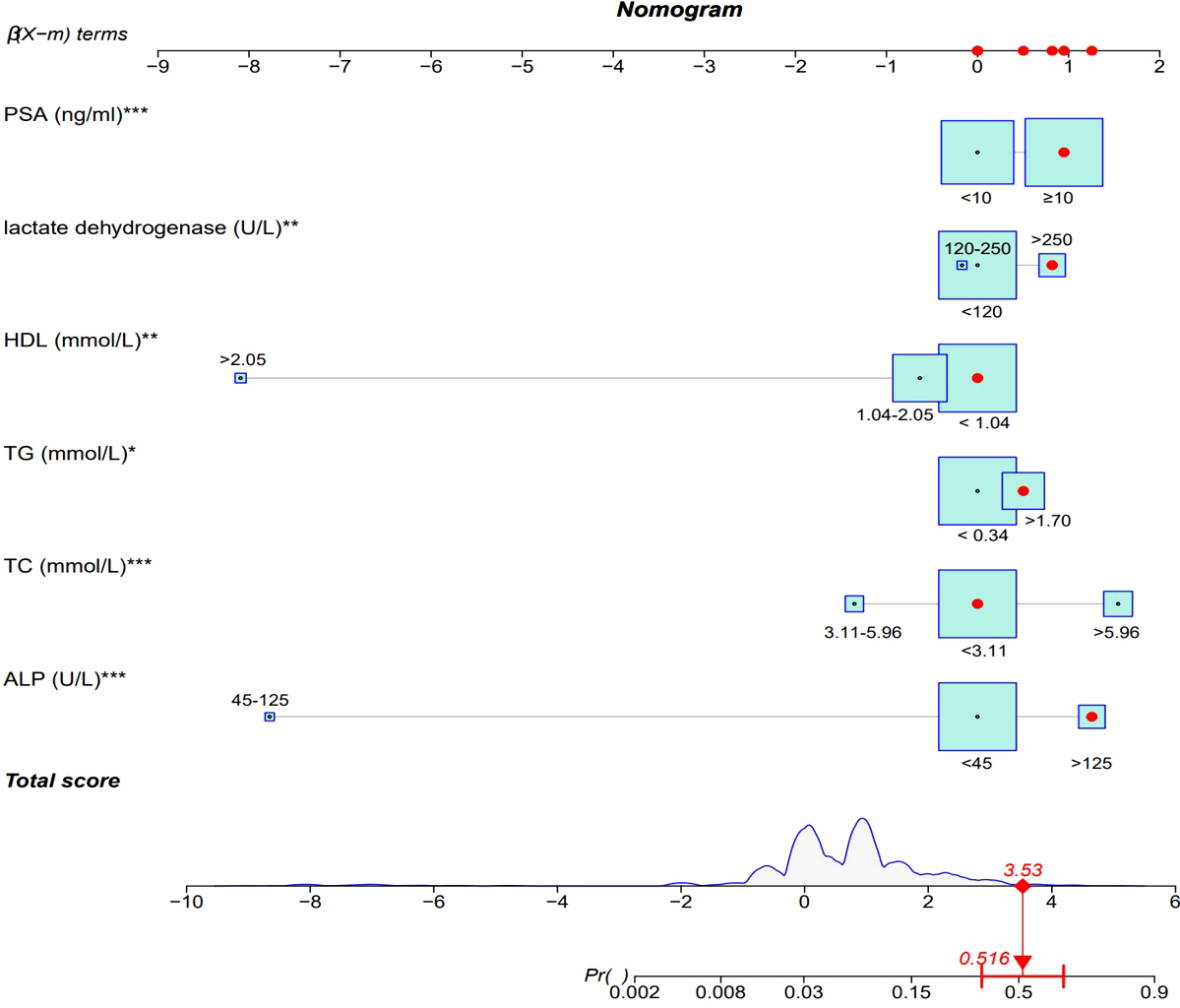
ALP, alkaline phosphatase; TC, total cholesterol; TG, triglycerides; HDL, high-density lipoprotein; PSA, prostate specific antigen.

Binary logistic regression analysis was performed to screen the predictors of PCa in the Ga cohort. Univariate analysis revealed that low ALB (OR: 0.637, 95% CI: 0.454~0.995; P = 0.047), high ALP (OR: 4.120, 95% CI: 2.722 ~6.236; P<0.001), high TC (OR: 4.960, 95% CI: 3.344~7.358; P<0.001), high TG (OR: 1.938, 95% CI: 1.335~2.813; P<0.001), high LDH (OR: 2.484, 95% CI: 1.580 ~3.906; P<0.001), low HDL (OR: 0.518, 95% CI: 0.339~0.792; P = 0.002), high LDH (OR: 2.484, 95% CI: 1.580~3.906; P<0.001) and high PSA (OR: 2.678, 95% CI: 1.806~3.971; P<0.001) were significantly associated with the diagnosis of IDC-P. (**S-Table II**) Variables that were significantly associated with diagnosis of IDC-P in univariate analysis were subjected to multivariate analysis. It was found that ALP, TC, TG, HDL, LDH and PSA were independent predictors of IDC-P (**Table 2**). Therefore, a predictive model containing ALP, TC, TG, HDL, LDH and PSA was established (**Figure 2**).

**Table 2:** Univariate and multivariate analysis for screening the predictors of outcomes of IDP-C.

	Univariate model			Multivariate model			
	OR	95%CI	P	B	OR	95%CI	P
ALB(L)	0.673	0.454~0.995	0.047	-0.189	0.828	1.388~3.839	0.392
ALP(H)	4.120	2.722 ~6.236	<0.001	1.267	3.549	2.241~5.621	<0.001
TC(H)	4.960	3.344~7.358	<0.001	1.531	4.624	3.011~7.101	<0.001
TG(H)	1.938	1.335~2.813	<0.001	0.477	1.611	1.060~2.448	0.026
LDH(H)	2.484	1.580 ~3.906	<0.001	0.836	2.308	1.388~3.839	0.001
HDL(L)	0.518	0.339~0.792	0.002	-0.606	0.546	0.343~0.867	0.010
PSA(H)	2.678	1.806~3.971	<0.001	0.943	2.567	1.698~3.881	<0.001

ALB, albumin; ALP, alkaline phosphatase; TC, total cholesterol; TG, triglyceride; HDL, high density lipoprotein; L,low group; H, high group.

**Figure 3:**
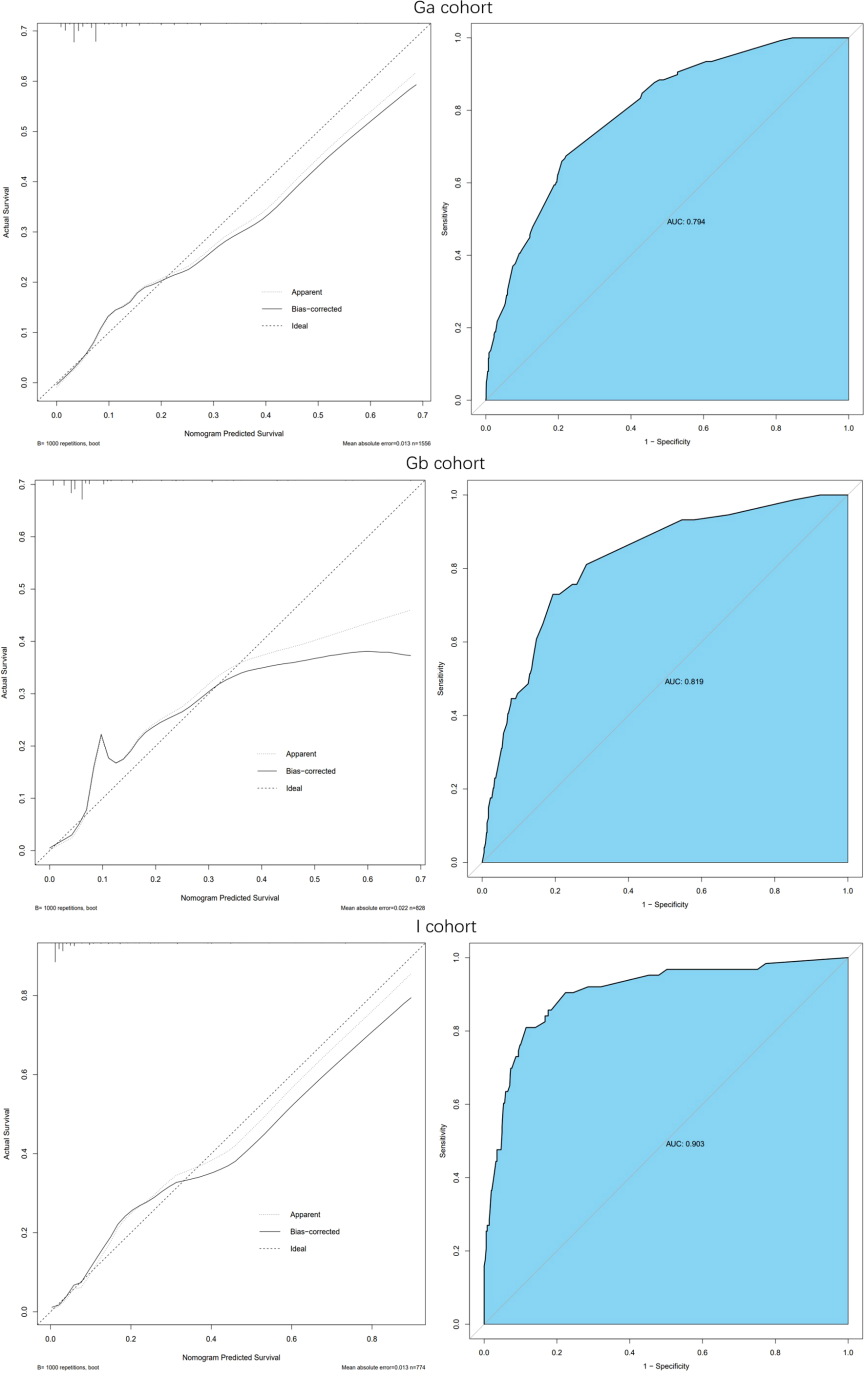
Calibration curve and ROC curve: Discrimination of the nomogram was evaluated by the ROC curve, AUC=0.794 in Ga cohort, AUC=0.819 in Gb cohort and AUC=0.903 in I cohort; Calibration curves illuminate the agreement between the predicted risks of IDP-C and the observed incidence of IDP-C. The dotted line represents an ideal flawless model.

Model verification revealed that it had excellent reproducibility (AUC=0.794). Moreover, both internal and external validations revealed that the predictive nomogram had a good discriminative power (AUC_Gb_ = 0.819; AUC_I_=0.903). Calibration curves showed good agreements between the predicted and observed incidence of IDC-P in the three cohorts (**Figure 3**).

### Decision curve analysis

**Figure 4:**
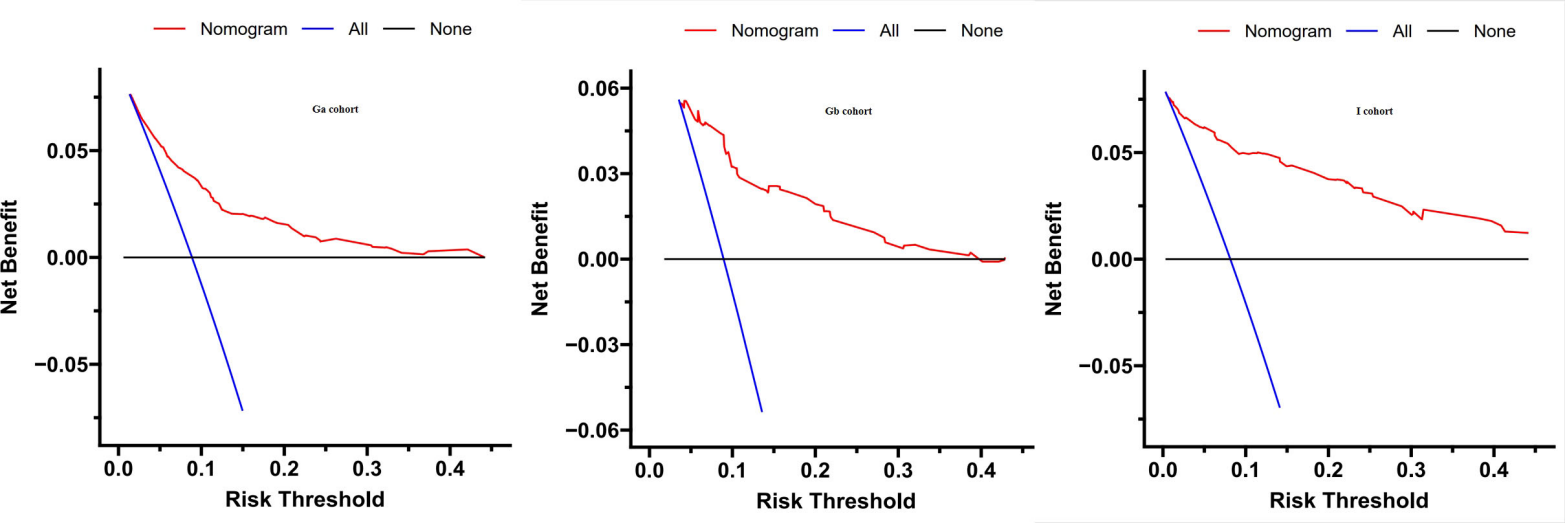
Decision curve analysis was prformed to estimate the clinical usefulness of the nomogram (IDP-C). The quantified net benefits were measured at different threshold probabilities. The y-axis denotes the standardized net benefit, while the x-axis denotes the threshold probabilities. The red line represents our nomogram, the blue line represents the condition that all patients have IDP-C, and the black line represents the condition that none has IDP-C.

To assess the clinical usefulness of our nomogram, a DCA curve was drawn. The DCA curve showed that the detection rate of IDP-C patients was effectively increased by our prediction model, These findings imply that the model can be used for early diagnosis of IDC-P (**Figure 4**).

## Discussion

With almost 1.4 million new cases and 375,000 deaths worldwide, prostate cancer was the second most frequent cancer and the fifth leading cause of cancer-associated deaths among men in 2020 (1). Clinically, IDC-P is a biologically aggressive pathological form of PCa that has poor prognostic outcomes. However, IDC-P in the absence of other prostate cancer pathological types is rare on biopsy, 27 cases out of a denominator of approximately 45 000 prostate needle biopsy consults (0.06%) (20). Pathologically, IDC-P is often accompanied by prostatic acinar adenocarcinoma (PAA) (7–10).

Since the early signs and symptoms of IDC-P tend to hide and clinicians as well as pathologists lack detailed knowledge and understanding of the pathological and clinical aspects of the disease, the possibility of a missed or delayed diagnosis cannot be ruled out. The presence of IDC-P indicates a poor prognostic outcomes for PCa. Therefore, early detection of IDC-P and formulation of an effective treatment plan is of clinical significance (21–25).

Even though there are no clear treatment guidelines for IDC-P, for localized prostate cancer, whether IDC-P or other pathological types, radical prostatectomy is the best choice. However, patients with metastatic IDC-P, IDC-P in the mCRPC stage, or patients with IDC-P who have lost the opportunity for surgery are treated differently from patients with prostate adenocarcinoma. Transcriptome sequencing of tumor lesions from the same patient after neoadjuvant hormonal therapy (NHT) revealed that NHT treatment significantly inhibited AR pathway activities in adenocarcinoma, but had less effects on AR signaling activities in IDC-P. Therefore, IDC-P may be resistant to classical endocrine therapy, but is sensitive to abiraterone therapy (12, 26–28).

Compared with other pathological types of tumors, IDC-P has a higher HRD score, suggesting that IDC-P has molecular characteristics of homologous recombination deficiency. Thus, IDC-P patients are more likely to carry the BRCA2 gene or BRCA-like molecular alterations. These molecular characteristic changes suggest that PARP inhibitors, represented by olaparib, are among the therapeutic options for improving the clinical prognosis of IDC-P positive patients (29).

Given the different treatment options for IDC-P and prostate adenocarcinoma, early diagnosis of IDC-P is necessary. Various models have been developed to predict PCa outcomes in the Chinese population (30, 31). Recently, Chen et al. (32). constructed the Chinese Prostate Cancer Consortium Risk Calculator (CPCC-RC) that is based on prostate-specific antigen (PSA), age, prostate volume, free to total PSA (fPSA-to-tPSA) rate, and digital rectal examination (DRE) to predict the initial prostate biopsy. However, the prediction models for IDP-C have not been established.

In this study, the risk factors (high TC, high TG, and low HDL) are all biochemical indicators associated with obesity in our study. Therefore, we postulated that IDC-P may be associated with the obesity status of patients. Excess fat mass is characterized by low-level chronic inflammation, which results in abnormal secretion of adipokines, leading to disrupted immune responses and other metabolic irregularities (33). This state leads to decreased cancer cell apoptosis and increased cancer cell growth and migration. Thus, these multiple factors may work in tandem to create a favorable tumor microenvironment for PCa cell growth in the catheter (34). Obesity has also been associated with insulin and sex hormone levels as well as the insulin-like growth factor axis. Perhaps, in this microenvironment, PCa cells proliferate in the normal prostate catheter and transform to IDC-P (35).

This study has several limitations. First, since it involved three hospitals in Zhejiang Province, China, it may not be reproducible in less experienced medical centers or other countries. Secondly, several indicators, such as TMN stage and PI-RADS were not included due to the irretrievably missing values and variance in measuring instruments. Thirdly, although we followed a set of inclusive and exclusive criteria during data collection, bias was inevitable, especially due to the independence of each hospital, but this also shows that our model has good universality. Moreover, prospective validation is required forour model.

## Conclusion

In summary, we established a clinical predictive nomogram consisting of ALP, TC, TG, HDL, LDH and PSA with excellent reproducibility and generalizability. This model provides a novel calculator for predicting the diagnosis of IDC-P and different treatment options for patients at an early stage.

## Data availability statement

The raw data supporting the conclusions of this article will be made available by the authors, without undue reservation.

## Ethics statement

The studies have been performed in accordance with the Declaration of Helsinki, and the participants were reviewed and approved by the Ethics Committee of Zhejiang Provincial People’s Hospital, Quzhou People’s Hospital and Jiaxing Second People’s Hospital. All study participants were informed about the planned procedure and signed informed consent. All methods were performed in accordance with the relevant guidelines and regulations.

## Author contributions

Conceptualization, YY; Data curation, LW, ZT, and QZ; Methodology, YY; Project administration, YY; Visualization, YY and WZ; Writing – original draft, YY and WZ; Writing – review & editing, YY, YB, and DZ. All authors contributed to the article and approved the submitted version.
