# Supplementary material for: Construction and validation of a clinical predictive nomogram for intraductal carcinoma of the prostate based on Chinese multicenter clinical data
Source: Front Oncol. 2022 Dec 15;12:1074478. doi: 10.3389/fonc.2022.1074478 (PMC9798232; doi:10.3389/fonc.2022.1074478)
Supplement: Supplementary file 1 [file DataSheet_1.docx]

S-Table I

|  | **Levene test** | | **mean T-test** | | | |
| --- | --- | --- | --- | --- | --- | --- |
|  | **F** | **significance** | **t** | **Sig** | **95%CI** | |
|  |  |  |  |  | **floor** | **upper** |
| age | 0.17 | 0.68 | 0.484 | 0.628 | -0.508 | 0.842 |
| kg | 0.31 | 0.58 | -1.882 | 0.060 | -1.590 | 0.033 |
| height | 0.34 | 0.56 | -1.064 | 0.287 | -0.007 | 0.002 |
| BMI | 0.63 | 0.43 | -1.737 | 0.082 | -0.481 | -0.029 |
| tpsa | 2.48 | 0.12 | -1.226 | 0.220 | -23.414 | 5.39 |
| ALB | 0.06 | 0.81 | 0.037 | 0.971 | -0.381 | 0.396 |
| GLB | 0.24 | 0.63 | 1.295 | 0.195 | -0.128 | 0.627 |
| ALT | 4.31 | 0.06 | 1.438 | 0.151 | -0.442 | 2.868 |
| AST | 0.24 | 0.63 | 0.120 | 0.904 | -1.109 | 1.254 |
| GGT | 0.48 | 0.22 | -0.922 | 0.356 | -5.914 | 2.130 |
| ALP | 0.06 | 0.83 | 0.096 | 0.924 | -15.692 | 17.305 |
| Tbil | 0.23 | 0.64 | 0.428 | 0.668 | -0.343 | 0.535 |
| bile acids | 0.32 | 0.58 | 0.773 | 0.439 | -0.316 | 0.729 |
| GLU | 0.08 | 0.93 | -0.076 | 0.940 | -0.142 | 0.131 |
| urea | 0.55 | 0.46 | -0.535 | 0.593 | -0.423 | 0.242 |
| creatinine | 0.68 | 0.41 | 0.083 | 0.934 | -3.348 | 3.645 |
| uric acid | 0.43 | 0.51 | -0.675 | 0.500 | -10.340 | 5.044 |
| TC | 0.00 | 0.99 | 0.125 | 0.901 | -0.804 | 0.91 |
| TG | 0.09 | 0.76 | -0.151 | 0.880 | -0.457 | 0.392 |
| HDL | 0.10 | 0.75 | 0.634 | 0.526 | -0.017 | 0.033 |
| LDL | 0.05 | 0.82 | 1.444 | 0.149 | -0.017 | 0.114 |
| L-D | 0.09 | 0.76 | -0.170 | 0.865 | -7.937 | 6.673 |
|  | | | | | | |

S-Table II

|  | B | SE | wald | df | sig | EXP | EXP(95%) | | | |
| --- | --- | --- | --- | --- | --- | --- | --- | --- | --- | --- |
|  |  |  |  |  |  |  | floor | | upper | |
| BMI(H) | 0.229 | 0.180 | 1.625 | 1 | 0.202 | 1.258 | | 0.884 | | 1.789 |
| diabetes | -0.122 | 0.277 | 0.194 | 1 | 0.660 | 0.885 | | 0.514 | | 1.525 |
| hypertension | 0.325 | 0.179 | 3.301 | 1 | 0.069 | 1.384 | | 0.975 | | 1.965 |
| CAD | -0.680 | 0.467 | 2.119 | 1 | 0.145 | 0.506 | | 0.203 | | 1.266 |
| smoke | -0.106 | 0.187 | 0.324 | 1 | 0.569 | 0.899 | | 0.623 | | 1.297 |
| Drink | 0.037 | 0.190 | 0.037 | 1 | 0.847 | 1.037 | | 0.714 | | 1.507 |
| PSA(H) | 0.985 | 0.201 | 24.014 | 1 | 0.000 | 2.678 | | 1.806 | | 3.971 |
| ALB(L) | -0.397 | 0.200 | 3.931 | 1 | 0.047 | 0.673 | | 0.454 | | 0.995 |
| GLB(L) | -0.331 | 1.039 | 0.101 | 1 | 0.750 | 0.718 | | 0.094 | | 5.505 |
| GLB(H) | -18.894 | 7882.490 | 0.000 | 1 | 0.998 | 0.000 | | 0.000 | | 0.001 |
| ALT(L) | 0.106 | 0.380 | 0.078 | 1 | 0.780 | 1.112 | | 0.528 | | 2.343 |
| ALT(H) | 0.180 | 0.642 | 0.079 | 1 | 0.779 | 1.197 | | 0.340 | | 4.215 |
| AST(L) | 0.686 | 0.429 | 2.558 | 1 | 0.110 | 1.985 | | 0.857 | | 4.601 |
| AST(H) | -0.225 | 0.834 | 0.073 | 1 | 0.788 | 0.799 | | 0.156 | | 4.098 |
| GGT(L) | -18.908 | 17974.843 | 0.000 | 1 | 0.999 | 0.000 | | 0.000 | | 0.001 |
| GGT(H) | -0.294 | 0.303 | 0.943 | 1 | 0.331 | 0.745 | | 0.412 | | 1.349 |
| ALP(L) | -18.647 | 8987.421 | 0.000 | 1 | 0.998 | 0.000 | | 0.000 | | 0.001 |
| ALP(H) | 1.416 | 0.211 | 44.827 | 1 | 0.000 | 4.120 | | 2.722 | | 6.236 |
| Tbil(H) | 0.295 | 0.366 | 0.651 | 1 | 0.420 | 1.344 | | 0.656 | | 2.752 |
| BA(H) | 0.029 | 0.407 | 0.005 | 1 | 0.944 | 1.029 | | 0.464 | | 2.284 |
| GLU(L) | -18.932 | 11147.524 | 0.000 | 1 | 0.999 | 0.000 | | 0.000 | | 0.001 |
| GLU(H) | -0.259 | 0.234 | 1.223 | 1 | 0.269 | 0.772 | | 0.488 | | 1.221 |
| Urea(L) | -0.424 | 1.037 | 0.167 | 1 | 0.683 | 0.654 | | 0.086 | | 4.995 |
| Urea(H) | -0.377 | 0.302 | 1.558 | 1 | 0.212 | 0.686 | | 0.380 | | 1.240 |
| UC(H) | -0.339 | 0.525 | 0.416 | 1 | 0.519 | 0.713 | | 0.255 | | 1.995 |
| Uric acid(L) | -0.021 | 0.534 | 0.002 | 1 | 0.968 | 0.979 | | 0.344 | | 2.786 |
| Uric acid(H) | -0.291 | 0.252 | 1.337 | 1 | 0.248 | 0.747 | | 0.456 | | 1.224 |
| TC(L) | -1.664 | 1.013 | 2.698 | 1 | 0.100 | .189 | | 0.026 | | 1.379 |
| TC(H) | 1.601 | 0.201 | 63.372 | 1 | 0.000 | 4.960 | | 3.344 | | 7.358 |
| TG(H) | 0.662 | 0.190 | 12.126 | 1 | 0.000 | 1.938 | | 1.335 | | 2.813 |
| HDL(L) | -0.658 | 0.216 | 9.231 | 1 | 0.002 | 0.518 | | 0.339 | | 0.792 |
| HDL(H) | -19.067 | 8770.825 | 0.000 | 1 | 0.998 | 0.000 | | 0.000 | | 0.001 |
| LDL(L) | -18.851 | 3361.105 | 0.000 | 1 | 0.996 | 0.000 | | 0.000 | | 0.001 |
| LDL(H) | 0.307 | 0.180 | 2.895 | 1 | 0.089 | 1.359 | | 0.954 | | 1.936 |
| lactate dehydrogenase (L) | -0.543 | 1.030 | 0.278 | 1 | 0.598 | 0.581 | | 0.077 | | 4.372 |
| lactate dehydrogenase (H) | 0.910 | 0.231 | 15.536 | 1 | 0.000 | 2.484 | | 1.580 | | 3.906 |

## L-low group；H-high group；

S-Table III

|  | **low-group** | **standard group** | **high-group** |
| --- | --- | --- | --- |
| BMI (kg/m^2^) | - | ≤24.00 | ＞24.00 |
| PSA(ng/mL) | - | ≤10.00 | ＞10.00 |
| ALB(g/L) | ＜40.00 | 40.00-55.00 | ＞55.00 |
| GLB(g/L) | ＜20.00 | 20.00-44.00 | ＞44.00 |
| ALT(U/L) | ＜7.00 | 7.00-40.00 | ＞40.00 |
| AST(U/L) | ＜13.00 | 13.00-35.00 | ＞35.00 |
| GGT(U/L) | ＜7.00 | 7.00-45.00 | ＞45.00 |
| ALP(U/L) | ＜50.00 | 50.00-135.00 | ＞135.00 |
| Tbil(umol/L) | ＜3.40 | 3.40-24.00 | ＞24.00 |
| BA(umol/L) | - | ≤15.00 | ＞15.00 |
| GLU(umol/L) | ＜3.92 | 3.92-6.16 | ＞6.16 |
| Urea(umol/L) | ＜2.60 | 2.60-7.50 | ＞7.50 |
| UC(umol/L) | ＜41.00 | 41.00-73.00 | ＞73.00 |
| Uric acid(umol/L) | ＜155.00 | 155.00-357.00 | ＞357.00 |
| TC(mmol/L) | ＜3.11 | 3.11-5.96 | ＞5.96 |
| TG(mmol/L) | ＜0.34 | 0.34-1.70 | ＞1.70 |
| HDL(mmol/L) | ＜1.10 | 1.10-2.25 | ＞2.25 |
| LDL(mmol/L) | ＜2.10 | 2.10-3.10 | ＞3.10 |
| LDH (U/L) | ＜120.00 | 120.00-250.00 | ＞250.00 |
